# Effect of Cross-Linking Cations on In Vitro Biocompatibility of Apple Pectin Gel Beads

**DOI:** 10.3390/ijms232314789

**Published:** 2022-11-26

**Authors:** Sergey Popov, Nikita Paderin, Elizaveta Chistiakova, Dmitry Ptashkin, Pavel A. Markov

**Affiliations:** 1 Institute of Physiology of Federal Research Centre “Komi Science Centre of the Urals Branch of the Russian Academy of Sciences”, 50 Pervomaiskaya Str., 167982 Syktyvkar, Russia; 2 National Medical Research Center of Rehabilitation and Balneology of the Ministry of Health of the Russian Federation, St. Novy Arbat, 32, 121099 Moscow, Russia

**Keywords:** apple pectin, calcium, zinc, iron, and aluminum cations, gel beads, swelling, protein adsorption, complement activation, cytokine production, cytotoxicity

## Abstract

The study aimed to compare the in vitro biocompatibility of pectin gels formed by different cross-linking cations. Hydrogel beads named CaPG, ZnPG, FePG, and AlPG were prepared from 4% solutions of apple pectin using ionotropic gelling with CaCl_2_, ZnCl_2_, FeCl_3_, and AlCl_3_, respectively. Cations influenced the gel strength of the wet gel beads in the following order (least strong) Ca^2+^ < Zn^2+^ < Fe^3+^~Al^3+^ (most strong). The swelling degree of the CaPG beads after 24 h of incubation in the RPMI-1640 medium was 104%, whereas the ZnPG, FePG, and AlPG beads swelled by 76, 108, and 134%, respectively. The strength of the pectin gel decreased significantly after incubation in the RPMI-1640 medium for 24 h, regardless of the cross-linking cation, although the FePG beads remained the strongest. All the pectin beads adsorbed serum proteins to a low degree, however the serum protein adsorption by the ZnPG and FePG beads (1.46 ± 0.87 and 1.35 ± 0.19 µg/mm^2^) was more than the CaPG and AlPG beads (0.31 ± 0.36 and 0.44 ± 0.25 µg/mm^2^). All the pectin beads reduced the production of TNF-α and IL-10 by hPBMCs in response to LPS stimulation. The IL-1β response of cells to LPS was significantly reduced by the CaPG, ZnPG, and FePG beads, whereas the AlPG beads enhanced it twofold. The CaPG, FePG, and AlPG beads had no cytotoxicity. The viability of hPBMCs and human fibroblasts incubated with ZnPG beads was 5.3 and 7.2%, respectively. Thus, the use of different cross-linking cations changed the properties of the pectin gel, which is important for biocompatibility.

## 1. Introduction

Pectin is an anionic polysaccharide found in the cell wall and the intracellular space of most flowering plants. Pectin is mainly made up of 1,4-linked *α-D*-galacturonic acid (GalA) residues, where carboxyl groups of GalA may be methyl-esterified and/or acetyl-esterified. In the branched areas of pectin, side chains composed of arabinose and galactose residues, predominantly, link to rhamnose residues inserted in the galacturonic backbone [1]. Depending on the degree of methyl esterification (DM), pectins are divided into high-methyl esterified pectins (HMP) with a DM value > 50% and low-methyl esterified pectins (LMP) with a DM < 50%. HMP can form physical gels at pH < 3.5 in the presence of co-solutes, and LMP can form gels in the presence of multivalent cations in a wide pH range [2]. Gelling capacity and low production costs ensure the wide use of pectin in the food and pharmaceutical industries [3,4]. In the cation-induced gelation of LMP, divalent and trivalent cations interact with the carboxylic groups of GalA to form “egg-box” junction zones, starting the dimerization of two slightly shifted chains and the subsequent aggregation of these dimers to form a gel network [2,5,6].

Pectin gels are promising biomaterials for wound and surgery dressings, tissue engineering, and biosensors. The uncomplicated release of drug molecules at high pH, antibacterial activity, and maintenance of an acidic environment are among the advantages of biomaterials based on the three-dimensional structure formed by LMP and cation bridges for wound healing. Their cytocompatibility regarding different types cells and the tunable mechanical properties of pectin gels contribute to their application as tissue engineering scaffolds [7].

It is important to note that calcium chloride is a cross-linker commonly used to prepare macroscopic gels of pectin in biomaterials. Besides calcium ions, other divalent or trivalent cations may form LM pectin gels. However, the influence of other cross-linking cations on the properties of pectin gel has been most thoroughly studied in the development of oral drug delivery systems, but not in tissue engineering materials. Various researchers reported the superiority of Zn^2+^ over Ca^2+^ in showing improved stability in the upper gastro-intestinal tract and slower drug release [8]. In the study by Sarioglu E. et al. [9], zinc-containing hydrogel for wound dressing showed better controlled drug release behavior and antibacterial activity than Ca^2+^-cross-linked hydrogel. It was shown that zinc and calcium interact with pectin chains differently. According to [10], zinc ions interact with both the carboxyl and the hydroxyl groups of pectin, while calcium ions interact only with carboxyl groups resulting in galacturonate chains that are more loosely associated with each other in the presence of Ca^2+^ than with Zn^2+^ ions. A lower chain mobility and thus a lower flexibility produces a stronger Zn^2+^–pectin gel matrix compared to Ca^2+^–pectin one. Trivalent cations, namely Fe^3+^ and Al^3+^, were used as an alternative cross-linker produce a pectinate network as reported in some studies [11,12]. For example, Al^3+^ ion cross-linking provided better control of the drug release rate than Ca^2+^ [13] and Zn^2+^ ions [14]. In the study by Wu X. et al. [15], pectin–Fe^3+^/poly (acrylamide-co-stearyl methacrylate) hydrogels supported the adhesion and proliferation of mouse chondrocytes. The dynamic nature of the metal-coordination bond allows for extra functionality of Fe^3+^–pectin gels. Specifically, the photoreduction of Fe^3+^ to ferrous ions mediates the gel–sol transition in light-responsive Fe^3+^−pectin coordination hydrogels for controlled delivery [16]. Magnesium chlorides, as a cross-linking agent, have been shown to form a scaffold of low stability [17].

In general, pectin-containing scaffolds are biocompatible [7,18]. However, the biocompatibility of pectin gels formed by different cross-linking cations has not been compared yet. The biocompatibility of bio-engineered material when it encounters body fluids is determined by the extent of cellular attachment, migration, and proliferation occurring on their surface as a result of the so-called foreign-body response (FBR). The initial stages of FBR include serum protein adsorption and the complement activation, adhesion, and activation of inflammatory leukocytes [19]. FBR can be alleviated or regulated by adjusting the structures, including the chemical and physical structure of the hydrogels. The physical-chemical properties of hydrogels, e.g., their hardness, elasticity, swelling, degradation, etc., determine the fate of cells on the surface or inside the hydrogel and, therefore, can regulate cell behavior and promote tissue regeneration [20]. In the present study, we hypothesized that the type of cation cross-linker would affect the biocompatibility of the pectin gel because the type of cross-linking cation is an important determinant for the pectin gelling mechanism and the properties of pectin gel.

The study aimed to compare the mechanical and swelling properties and the biocompatibility of gel beads prepared from apple pectin cross-linked with Ca^2+^, Zn^2+^, Fe^3+^, and Al^3+^ cations. For this, the serum protein and lipopolysaccharide (LPS) adsorption, complement activation, cytokine production by human peripheral blood mononuclear cells, and viability of human fibroblasts were determined in vitro to investigate the effect of pectin gel beads on the initial stages of FBR.

## 2. Results

### 2.1. Characterization of Pectin Gel Beads

**Figure 1:**
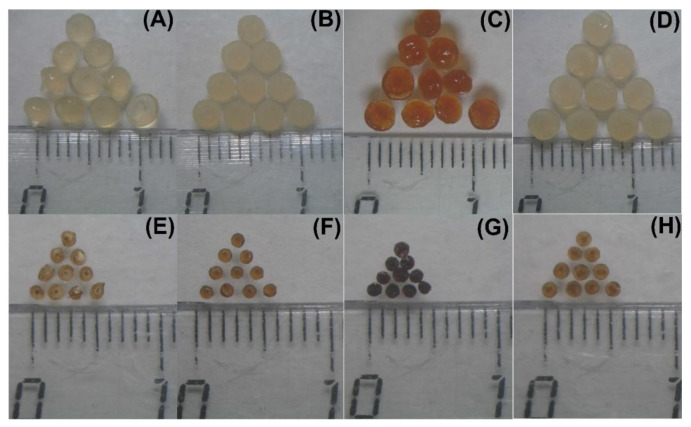
Photographs of wet (**A**–**D**) and dried (**E**–**H**) CaPG, ZnPG, FePG, and AlPG beads.

Hydrogel beads named CaPG, ZnPG, FePG, and AlPG were prepared from 4% solutions of apple pectin using ionotropic gelling with CaCl_2_, ZnCl_2_, FeCl_3_, and AlCl_3_, respectively. Images of the wet and dried gel beads are shown in Figure 1.

The wet ZnPG beads had the smallest diameter and surface area and were of the highest density among wet gel beads (Table 1). The surface area of the wet ZnPG beads was 15–21% lower than the CaPG, FePG, and AlPG beads. The density of the wet ZnPG beads was 24, 36, and 48% higher than CaPG, FePG, and AlPG beads, respectively. The ZnPG and AlPG beads were spherical, as evidenced by the sphericity factor (SR, Table 1), and their aspect ratio was close to 1.0. The CaPG and FePG beads were less spherical. The water content, measured by drying the wet beads to a constant weight at 25 °C, was 94.7–95.7%. The dried, similar to the wet, ZnPG beads had the smallest dimensions and the highest density among the dried gel beads (Table 1). The density of the dried CaPG beads was 47% lower than the ZnPG beads.

**Figure 2:**
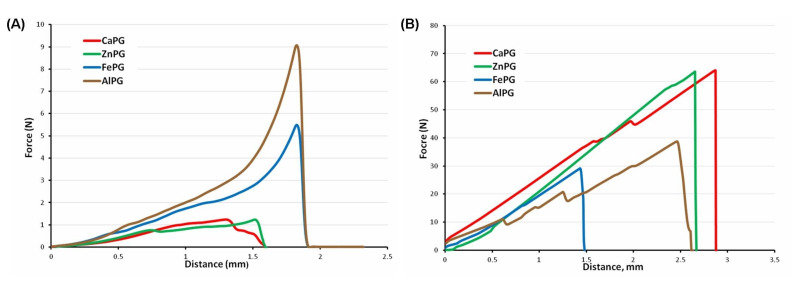
Mean (*n* = 10) force overtime curve of the wet (**A**) and dried (**B**) CaPG, ZnPG, FePG, and AlPG beads.

The mean force overtime curve obtained using the compression test of the gel beads is shown in Figure 2. The gel strength was determined as a force measured when the probe reached 0.5 mm after the start of sample compression. Cations influenced the gel strength of the wet gel beads in the following order (the weakest) Ca^2+^ < Zn^2+^ < Fe^3+^~Al^3+^ (strongest). The gel strength of the wet CaPG beads was 2.8 times lower than the wet FePG and AlPG beads (Table 1). However, the dried FePG and AlPG beads were the least strong among the dried beads, whereas the ZnPG beads were the strongest.

### 2.2. Swelling Studies

**Figure 3:**
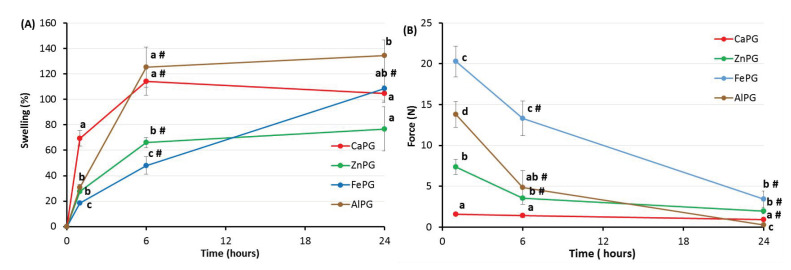
Swelling degree (**A**) and gel strength (**B**) of CaPG, ZnPG, FePG, and AlPG beads incubated for 24 h in RPMI-1640 medium supplemented with 10% FBS. The data are presented as the mean (*n* = 5). Different letters indicate significant differences between pectin gel beads prepared with different cations (*p* < 0.05). # *p* < 0.05 vs. previous time point.

The swelling of the dried pectin gel beads incubated for 24 h in RPMI-1640 medium supplemented with 10% FBS was investigated (Figure 3A). The CaPG and AlPG beads gradually swelled for 6 h to a level of ca. 120%, and then they maintained this bead size for 24 h. The ZnPG and FePG beads swelled more slowly; after 6 h of incubation, they increased in size by 66 and 48%, respectively. Then, the ZnPG beads kept their size at 176% of the original size, while the FePG beads continued to swell, so that they doubled in size compared to the original size after 24 h of incubation (Figure 3A).

After 24 h of incubation, the surface area of one gel bead was 19 ± 1, 15 ± 4, 22 ± 3, and 28 ± 3 mm^2^ for the CaPG, ZnPG, FePG, and AlPG beads, respectively.

After incubation for 1 h, the gel strength of the CaPG and ZnPG beads was at the level of 4 and 12% of the initial level of the dried beads’ gel strength (Table 1, Figure 3). The FePG and AlPG beads lost their gel strength to a lesser extent during the first hour of swelling than the CaPG and ZnPG beads did. The gel strength of the FePG and AlPG beads was at the level of 69 and 49% of the initial level of dried beads’ gel strength after incubation for 1 h in the RPMI-1640 medium (Table 1, Figure 3). A decrease in the gel strength accompanied the subsequent swelling of all the pectin gel beads, except for the CaPG ones. The FePG beads were the strongest, and the AlPG beads were the softest after 24 h of incubation in the RPMI-1640 medium (Figure 3).

### 2.3. Serum Protein and Lipopolysaccharide (LPS) Adsorption

**Figure 4:**
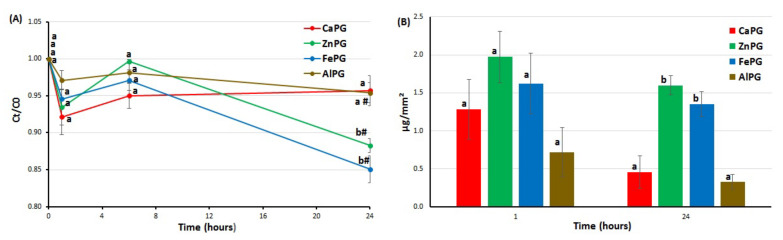
The serum protein adsorption by CaPG, ZnPG, FePG, and AlPG beads incubated for 24 h in RPMI-1640 medium supplemented with 10% FBS. Adsorption is expressed as the relative protein concentration Ct/C0 (**A**) and as protein adsorption per unit surface area (**B**). The data are presented as the mean ± SD (*n* = 5). Different letters indicate significant differences between pectin gel beads prepared with different cations (*p* < 0.05). # *p* < 0.05 vs. previous time point. C0 and Ct are the initial and remaining concentrations of protein in solution at different time points after incubation, respectively.

The dried CaPG, ZnPG, FePG, and AlPG beads were incubated for 24 h in RPMI-1640 medium supplemented with 10% FBS to study their protein-adsorption capacity. The initial serum protein content in the RPMI-1640 medium supplemented with 10% FBS was 5.2 ± 0.01 mg/mL. The protein concentration in the medium did not change significantly as a result of the immersion of all the pectin gel beads, except for the FePG beads (Figure 4A). Considering the change in the surface area of the beads during swelling, it was found that ZnPG and FePG adsorbed 4–4.5 times more serum protein than the CaPG and AlPG beads after incubation for 24 h (Figure 4B).

**Figure 5:**
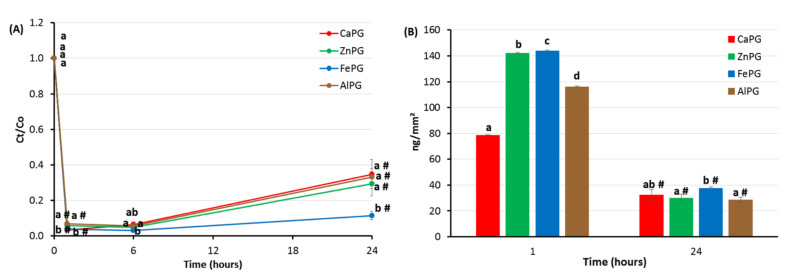
The lipopolysaccharide (LPS) adsorption by CaPG, ZnPG, FePG, and AlPG beads incubated for 24 h in RPMI-1640 medium supplemented with 10% FBS. Adsorption is expressed as the relative LPS concentration Ct/C0 (**A**) and as LPS adsorption per unit surface area (**B**). The data are presented as the mean ± SD (*n* = 5). Different letters indicate significant differences between pectin gel beads prepared with different cations (*p* < 0.05). # *p* < 0.05 vs. previous time point. C0 and Ct are the initial and remaining concentrations of LPS in solution at different time points after incubation, respectively.

The CaPG, ZnPG, FePG, and AlPG gel beads were co-incubated in the RPMI-1640 medium supplemented with 10% FBS and 10 μg/mL LPS. The LPS concentration decreased after 1 h in the incubation medium containing pectin gel beads (Figure 5A). Considering the change in the surface area of the beads during swelling, it was found that the ZnPG, FePG, and AlPG beads adsorbed 1.8, 1.8, and 1.5 times more LPS than the CaPG beads after incubation for 1 h (Figure 5B). Then, part of the adsorbed LPS apparently resorbed during the 24 h of incubation. The LPS adsorption expressed per unit surface area was 28–37 ng/mm^2^ for all the pectin gel beads, regardless of the cross-linking cation (Figure 5B).

### 2.4. Complement Activation

**Figure 6:**
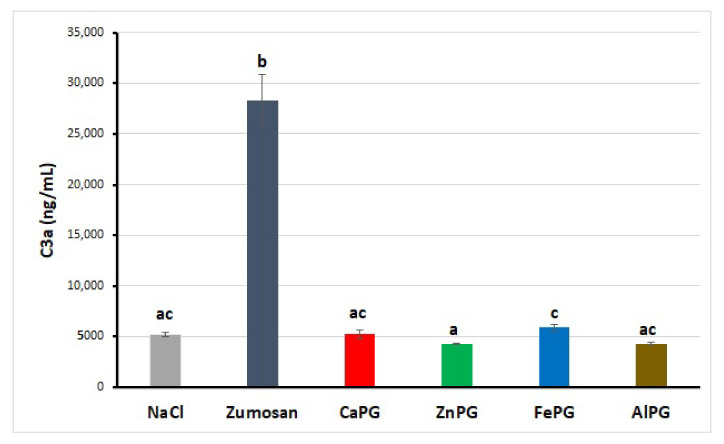
Effect of CaPG, ZnPG, FePG, and AlPG beads on C3a production in the whole blood in vitro. Results are presented as the mean ± SD (*n* = 6). Different letters indicate significant differences between pectin gel beads prepared with different cations (*p* < 0.05).

The release of the C3a complement component in human blood failed to change after co-incubation with CaPG, ZnPG, FePG, and AlPG beads and was similar to that of the negative control (NaCl) samples (Figure 6).

### 2.5. Cytokine Production by Human Peripheral Blood Mononuclear Cells (hPBMCs)

**Figure 7:**
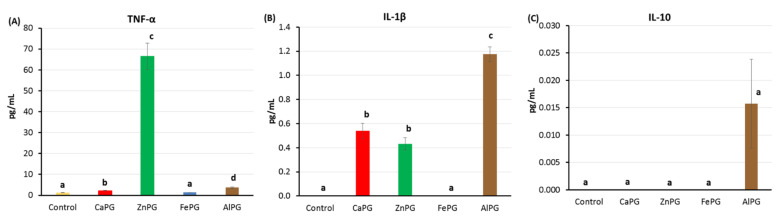
Effect of CaPG, ZnPG, FePG, and AlPG beads on tumor necrosis factor-α (TNF-α, (**A**)), interleukin-1β (IL-1β, (**B**)), and interleukin-10 (IL-10, (**C**)) production by non-stimulated human peripheral blood mononuclear cells (hPBMCs). Different letters indicate significant differences between pectin gel beads prepared with different cations (*p* < 0.05).

The level of the proinflammatory cytokines tumor necrosis factor-α (TNF-α), interleukin-1β (IL-1β), and anti-inflammatory cytokine interleukin-10 (IL-10) in the cell supernatant 24 h after incubation in hPBMCs suspension with pectin gel beads was measured as an indicator of inflammatory stimulation. In the LPS-free cell medium, the hPBMCs incubated with CaPG and AlPG beads produced TNF-α 1.7- and 2.8-fold compared to the control cells. The FePG beads failed to stimulate TNF-α production by hPBMCs, whereas the ZnPG beads enhanced production by 50 times that of the TNF-α level compared to the control (Figure 7A). The CaPG, ZnPG, and AlPG beads slightly stimulated IL-1β production in the absence of LPS (Figure 7B). The level of IL-10 was negligible regardless of the presence of pectin gel beads (Figure 7C).

**Figure 8:**
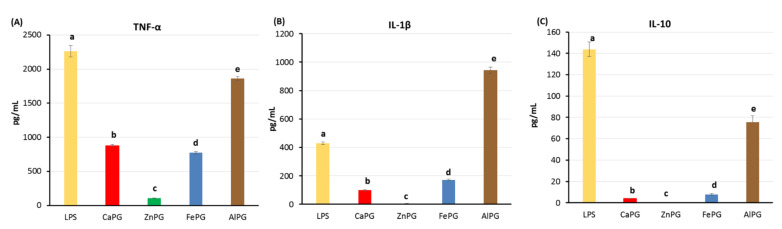
Effect of CaPG, ZnPG, FePG, and AlPG beads on tumor necrosis factor-α (TNF-α, (**A**)), interleukin-1β (IL-1β (**B**)), and interleukin-10 (IL-10, (**C**)) production by human peripheral blood mononuclear cells (hPBMCs) stimulated with LPS 2 µg/mL. Different letters indicate significant differences between pectin gel beads prepared with different cations (*p* < 0.05).

LPS (2 µg/mL) significantly stimulated the production of cytokines by hPBMCs. The LPS-stimulated cells produced 2262 ± 215, 429 ± 23, and 144 ± 18 pg/mL TNF-α, IL-1α, and IL-10, respectively. The cytokine response of cells to LPS was significantly reduced in the presence of pectin gel beads. LPS-stimulated TNF-α production was reduced to the maximum extent by the ZnPG beads, which decreased the level of TNF-α by 21 times (Figure 8A). The CaPG and FePG beads decreased the TNF-α level by 2.6 and 2.9 times, respectively, compared to the control cells. The AlPG beads had the least inhibiting effect on TNF-α production (Figure 8A). Similar to TNF-α, the production of IL-1β and IL-10 by the LPS-stimulated cells was suppressed by the ZnPG beads almost completely (Figure 8B,C). The CaPG and FePG beads decreased the IL-1β level by 4.3 and 2.5 times and decreased the IL-10 level by 35 and 18 times, respectively, compared to the control cells. The AlPG beads reduced the production of IL-10; however, they enhanced of the production of IL-1β by two-fold (Figure 8B,C).

### 2.6. Viability of hPBMCs

**Figure 9:**
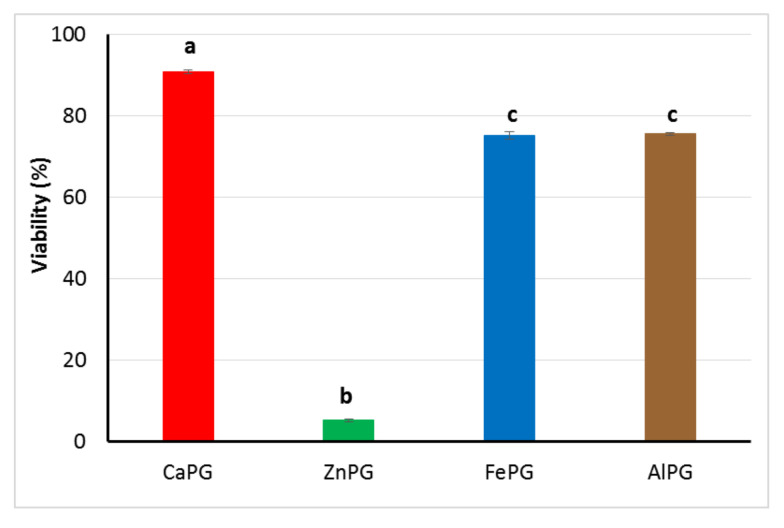
Effect of CaPG, ZnPG, FePG, and AlPG beads on viability of human peripheral blood mononuclear cells (hPBMCs). Control (cells in the presence of culture medium) corresponded to 100% viability. Different letters indicate significant differences between pectin gel beads prepared with different cations (*p* < 0.05).

To evaluate possible cytotoxic effects of pectin beads, their viability was determined by the live/dead cell viability test based on the simultaneous determination of live and dead cells using two different fluorescence dyes. Live cell dye easily penetrates intact, live cells, and intracellular esterase hydrolyzes the dye to produce a hydrophilic compound that is retained in the cell cytoplasm and emits an intense green fluorescence. Dead cell dye can only penetrate damaged cell membranes and then bind nucleic acid and emit a bright red fluorescence. As shown in Figure 9 and Figure S1B, The CaPG beads did not significantly affect the viability of hPBMCs. The cell cultures were also viable, with the percentage of live cells above 70% after 24 h incubation with the FePG and AlPG beads (Figure 9 and Figure S1D,E). However, the ZnPG beads were significantly toxic, decreasing the cell viability down to 5.3% of the viability level of the untreated cells incubated in a culture medium (Figure 9 and Figure S1C).

### 2.7. Viability of Human Fibroblasts

The effect of pectin beads on human fibroblasts was investigated using Annexin V and propidium iodide staining. Annexin V is a phospholipid-binding protein with a high affinity for membrane phosphatidylserine, which is exhibited on the plasma membrane of apoptotic cells. The membranes of dead and damaged cells are permeable to propidium iodide, whereas viable cells with intact membranes exclude propidium iodide.

**Figure 10:**
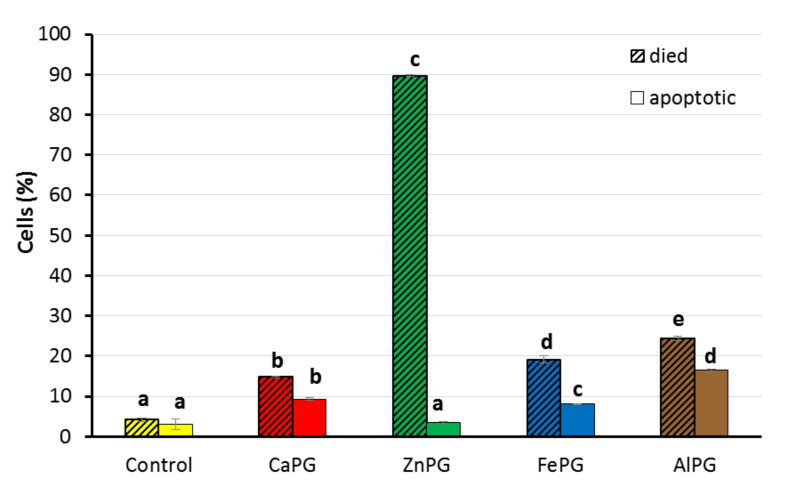
Effect of CaPG, ZnPG, FePG, and AlPG beads on viability of human fibroblasts. Control (cells in the presence of culture medium) corresponded to 100% viability. The bars shaded diagonally indicate the number of propidium-iodide-positive cells. Uniformly colored bars without shading indicate the number of Annexin-V-positive and propidium-iodide-negative cells Different letters indicate significant differences between pectin gel beads prepared with different cations (*p* < 0.05).

The number of FITC-Annexin-V-negative and propidium-iodide-negative, i.e., viable, fibroblasts, was 82.0 ± 1.6, 7.2 ± 0.1, 78.6 ± 2.8, and 63.7 ± 2.3% after incubation with the CaPG, ZnPG, FePG, and AlPG beads, respectively, when the number of viable cells in control was 100%. Most fibroblasts were propidium-iodide-positive after incubation with the ZnPG beads (Figure 10). The number of dead fibroblasts was 3.5, 4.6, and 5.7 times higher under treatment with the CaPG, FePG, and AlPG beads, respectively, than those in the untreated cell suspension (Figure 10). These dead fibroblasts may have included both cells that died as a result of a necrotic pathway and cells that suffered a loss of membrane integrity at the end stage of apoptosis. The number of Annexin-V-positive and propidium-iodide-negative fibroblasts, i.e., fibroblasts that were in early apoptosis, was 3.0 ± 3.6% in the control cell culture. The number of apoptotic cells increased by 3.1, 2.7, and 5.3 times after incubation with the CaPG, FePG, and AlPG beads, respectively (Figure 10). The ZnPG beads failed to change the number of apoptotic cells in comparison with the control cell culture.

As can be seen from the microscopic photograph (Supplementary Figure S2), the density of the fibroblast cell layer did not change significantly during the 24 h of incubation with the pectin gel beads. However, the cell body area of the fibroblasts was significantly reduced when incubated with the ZnPG, FePG, and AlPG beads compared to the control cells, indicating a lower level of cell-spreading on the surface. The area of the cell body of one fibroblast in the control and in the presence of the CaPG beads was 2893 ± 446 and 3047 ± 491 µm^2^, respectively. The area of the fibroblast’s body after incubation with the ZnPG, FePG, and AlPG beads was 1603 ± 271, 1969 ± 213, and 1003 ± 173 µm^2^, respectively.

## 3. Discussion

Calcium ions are mainly used as cross-linkers to obtain ionotropic pectin gels. However, other cations are increasingly being considered as an alternative to calcium ions in order to improve the physicochemical and functional properties of calcium–pectin gels. Zn^2+^ represents positively charged divalent ions, such as Ca^2+^, which may cross-link pectin polymer chains, forming a gel network. Particularly, Zn^2+^ ions are used as an alternative cross-linker because they produce a stronger pectinate network and show improved stability in the upper gastro-intestinal tract [8]. Zinc ions are added to biomaterials, especially for wound healing, due to their antibacterial action [9]. Transition metal ions such as Fe^3+^ ions are used as an alternative to Ca^2+^ cross-linkers to produce a pectinate network [12]. The coordination bond formed with transition metal ions creates a covalent contribution not seen with ions such as calcium. The dynamic nature of the metal-coordination bond imparts new mechanical properties to the materials that can be controlled by changing the metal-coordination environment [16]. Al^3+^ ion cross-linking provides better control of the drug release rate than Ca^2+^ [13] and Zn^2+^ ions [14].

It is known that the physicochemical properties of pectin gels depend on the cross-binding cation [12], and their biocompatibility, in turn, especially the initial stages of the foreign-body response, is determined by the physicochemical properties of the biomaterial [20]. In the present study, the type of cross-linking cation was found to influence the properties of the pectin gels, which determined their biocompatibility, such as the mechanical properties, swelling behavior, serum protein adsorption, complement activation, and inflammatory response.

### 3.1. Effect of Cross-Linking Cations on the Mechanical Properties and Swelling Behavior of PG Beads

**Figure 11:**
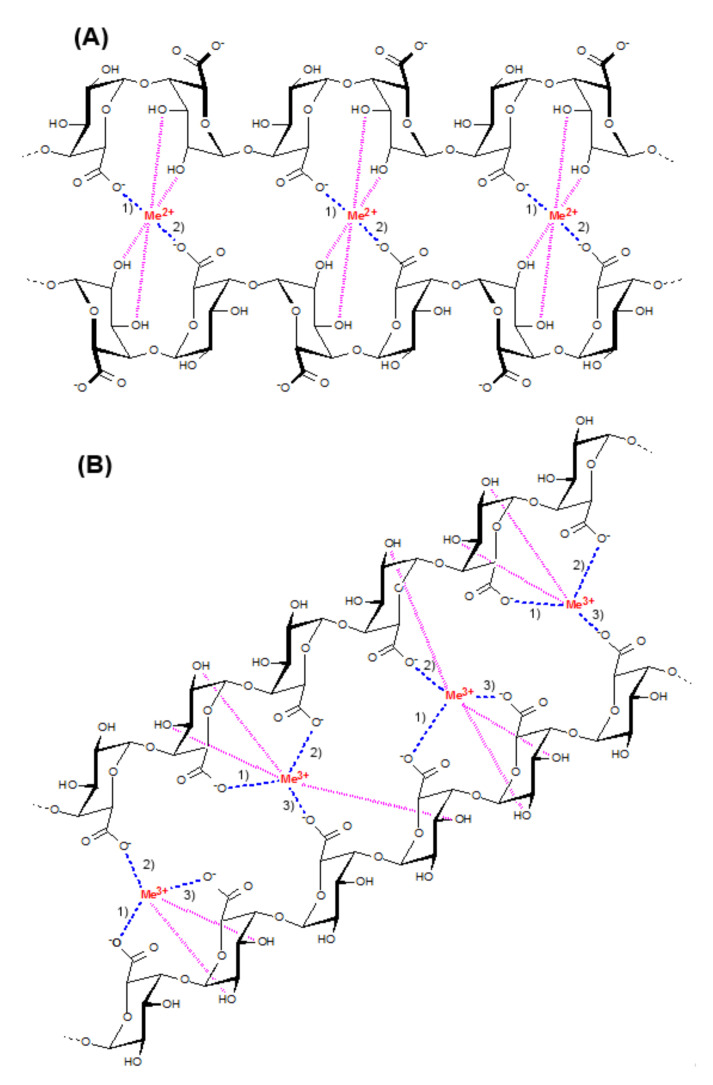
Mechanism presentation of the “egg-box” model of pectin gelation induced by divalent (Zn^2+^ and Ca^2+^) (**A**) and trivalent (Fe^3+^ and Al^3+^) (**B**) cations. Numbers 1, 2, and 3 indicate ionic bonding; pink dotted lines indicate hydrogen bonds.

The cations used in this study influenced the gel strength of the wet gel beads in the following order: Ca^2+^ < Zn^2+^ < Fe^3+^ < Al^3+^ (the strongest). LMP forms gels in the presence of divalent cations such as calcium and zinc ions by the so-called “egg box” mechanism [5,6]. A schematic representation of the “egg-box” model for junction zone formation in pectin calcium or pectin zinc gel is presented in Figure 11A. Pectin gelling by divalent cations is well known to involve two ionic links between the free carboxylic acid groups (COO^−^) of two neighboring pectin chains [2]. In addition to ionic bridges, egg-boxes are stabilized by hydrogen bonds and van der Waals interactions. In agreement with previous data, the ZnPG beads were stronger than the CaPG beads [10]. It was shown that zinc and calcium interacted with the pectin chains differently. According to [10], zinc ions interact with both the carboxyl and the hydroxyl groups of pectin, while calcium ions interact only with the carboxyl groups, resulting in galacturonate chains that are more loosely associated with each other in the presence of Ca^2+^ ions than with Zn^2+^ ions. A lower chain mobility and, thus, less flexibility, produces a stronger Zn^2+^–pectin gel matrix compared to a Ca^2+^–pectin one.

Trivalent Fe^3+^ or Al^3+^ ions have been suggested to form a stronger three-dimensional network due to the possibility of them forming an additional ionic bond between pectin chains, as shown in Figure 11B.

The swelling behavior was found to correspond to the gel strength of the dry pectin gel beads, so that the ZnPG beads swelled the least and the AlPG beads swelled the most. Previously, it was shown that the swelling of pectin gel depends on pH. Calcium–pectin gel beads collapsed in phosphate buffer solutions at pH 3.0 and swelled more at pH 7.4 and 8.0 than at pH 5.0 [18]. Pectin/acrylamide hydrogels in phosphate buffer solutions swelled more in a solution at pH 7.4 than at pH 1.2 and pH 5.4 [21,22]. The swelling of pectin gel at pH values higher than the pKa of D-GalA values (ca. 3.5) occurs because of electrostatic repulsion between pectin chains [23] and the exchange of cross-linking cations with monovalent ions such as Na^+^ [24]. In the present study, the CaPG beads swelled more than the ZnPG beads, and the AlPG beads swelled more than the FePG beads. Differences in the ions’ electronegativity may partially explain these results. Ca^2+^ cations are less electronegative (1.00) than Zn^2+^ (1.65) ones, and Al^3+^ ions are less electronegative (1.61) than Fe^3+^ (1.83) ones. It is assumed that a more electronegative element attracts electrons to itself more easily, which leads to stronger binding [12]. This suggests that Zn^2+^ and Fe^3+^ ions bind more strongly to pectin than Ca^2+^ and Al^3+^ ions, respectively. The swelled FePG beads remained the strongest, although their swelling behavior was close to that of the CaPG ones. An additional ionic bond in the egg-box structure of pectin gel may explain this result. It was demonstrated that a higher amount of Fe^2+^ could bind to pectin in comparison with Ca^2+^ or Zn^2+^ [25]. The AlPG beads swelled to the greatest degree and were the weakest both before and after incubation in RPMI-1640 medium at pH 7.4. The ionic radii of Al^3+^ ions are lower (50 pm) compared to those of Ca^2+^ (99 pm), Zn^2+^ (70 pm), and Fe^3+^ (70 pm) ions. Therefore, Al^3+^ ions may diffuse from the gel matrix faster than other ions, accelerating swelling. Thus, the degree of electronegativity and the size of the ion seemed to affect the swelling behavior of the pectin gel in addition to the features of the formation of ionic bonds in the egg-box structure.

### 3.2. Effect of Cross-Linking Cations on Protein Adsorption, Cytokine Production, and Cytotoxicity of PG Beads

Protein adsorption on the surface is one of the first events after biomaterial implantation that mediates the several biochemical reactions that ultimately define the biocompatibility of biomaterials (e.g., cell adhesion and cell proliferation) [26]. Nonspecific protein adsorption, or biofouling, remains a challenging problem in biomedical applications. A surface that adsorbs less than 5 ng/cm^2^ of protein is considered to exhibit “ultra-low fouling” [27]. The CaPG beads adsorbed 13 and 3 ng/cm^2^ of protein after 1 and 24 h of incubation in the RPMI-1640 medium supplemented with 10% FBS. Most polysaccharides, which are rich in hydroxyl groups and negatively charged, have demonstrated good protein resistance at a physiological pH [28,29]. Pectin hydrogel may prevent protein adsorption via the binding of water through both hydrogen bonding and ionic solvation. The absence of complement activation was consistent with the data on low protein adsorption on pectin gel particles. Indeed, the formation of an active C3a fragment requires the adhesion of the serum C3 protein on the foreign surface to initiate complement activation by the alternative pathway. In general, the low unspecific protein adsorption and lack of complement cascade stimulation indicated that the CaPG beads were biocompatible. Considering the change in the surface area of the beads during swelling, it was found that ZnPG and FePG adsorbed 4–4.5 times more serum protein than CaPG. In general, there are many factors affecting protein adsorption on a biomaterial’s surface, such as hydrophilicity, surface charge, and topography [20]. Proteins mainly interact with the surface of biomaterials through electrostatic interactions, van der Waals forces, and hydrogen bonds [27]. The lower ability to absorb water (swelling), resulting the lower hydrophilicity of the ZnPG beads, may partially explain the higher serum adhesion on the surface of the ZnPG beads compared to the CaPG beads. However, the swelling behavior of the FePG beads was close to that of the CaPG ones. A molecular mechanism that underlies the higher adhesion of serum proteins on the surface of the Fe^3+^-cross-linked pectin gel remains unexplored.

The stimulation of inflammatory leukocytes following nonspecific protein adsorption and the activation of complement proteins reduces the functionality of an implant and may cause its disintegration [30]. Blood neutrophils and monocytes, in particular, mediate interactions with biomaterials. Monocytes can differentiate into macrophages, which are important mediators of inflammation. Inflammatory activation of these cell types can ultimately lead to a subsequent adverse (usually catastrophic) response to biomaterial implantation [27]. Here, hPBMCs were incubated with the pectin gel beads, and the production of cytokines was measured 24 h after incubation. All the pectin beads significantly reduced TNF-α, IL-1β, and IL-10 production by hPBMCs stimulated with LPS, indicating an anti-inflammatory effect. This decrease may in part be because of a decrease in cytokine-producing cells, as the level of viable cells decreased after 24 h of incubation with the gel beads. About 10–25% of the hPBMCs died when co-incubated with the CaPG, FePG, and AlPG beads, thereby reducing the number of cells producing cytokines. Virtually all the hPBMCs died after 24 h of incubation with the ZnPG beads, showing a damaged cell membrane in the live/dead test. Cellular debris from dead cells appeared to stimulate the surviving LPS-unstimulated cells to produce a large amount of TNF-α. However, the functional response of cells to LPS was negligible after ZnPG bead treatment due to the meager number of living reactive cells. The anti-inflammatory effect of the pectin gel beads can also be explained by the partial adsorption of LPS, which reduced the number of LPS molecules available for cell receptor binding and cell activation. In addition, swelling and partial disintegration of the gel matrix during incubation could release polysaccharide chains with anti-inflammatory activity. Pectins have previously been shown to possess anti-inflammatory effects [31]. The main finding regarding the AlPG beads was their stimulating effect on IL-1β production by hPBMCs. The AlPG beads suppressed the production of TNF-α and IL-10 to the least extent compared to the CaPG, ZnPG, and FePG beads. For several decades, aluminum phosphate and aluminum hydroxide have been widely used as adjuvants in humans, representing an acceptable compromise between toxicity and adjuvanticity [32]. Previously, it was shown that aluminum salts released large amounts of IL-1β from hPBMCs, dendritic cells, and macrophages [33,34]. AlCl_3_ activated IL-1β signaling pathway in the hippocampus [35]. Alum-induced IL-1β production does not require intact Toll-like receptor (TLR) signaling but rather involves NLRP3 inflammasome activation [36]. Therefore, our results that showed that the AlPG beads did not stimulate TNF-α (TLR4-mediated cytokine), but only increased IL-1β levels, may demonstrate similar effects of Al^3+^ cross-linked pectin gel and aluminum adjuvants. However, the biocompatibility of the AlPG beads seemed to be lower than that of the CaPG beads because of inflammatory activation.

The cytocompatibility of pectin gel beads has been studied in human fibroblasts. The viability of fibroblasts was 64–82% after incubation with the CaPG, FePG, and AlPG beads. During FBR, stimulated monocytes/macrophages attract and activate fibroblasts to the surface of a biomaterial, which deposit collagen and other extracellular matrix proteins to form granulation tissue [19]. At the same time, fibroblasts are necessary for healing, synthesizing components of the intercellular substance. A high cytotoxicity of ZnPG beads was found, indicating that Zn^2+^ cross-linked pectin gel could interfere with the normal healing process, probably because of the toxicity of Zn^2+^ ions. For the first time, pectin gel was found to increase the number of apoptotic cells. The mechanism of the activation of the apoptotic pathway in fibroblasts by pectin gel remains unknown.

The interaction of the gel surface with serum proteins and cells is known to be determined not only by the mechanical properties of the gel itself but also, to a large extent, by the behavior of protein molecules and the cell membrane under the specific physicochemical conditions of interaction with gel material. Since the latter were not determined in the study, it is therefore difficult to establish a mechanism that explains the differences in the biocompatibility properties of gels cross-linked with different ions.

## 4. Materials and Methods

### 4.1. Preparation of Gel Beads

Apple pectin (AU701, Herbstreith and Fox (Nuremberg, Germany)) was dissolved in distilled water (40 mg/mL). The pectin solutions were extruded drop-wise using a pump (constant speed 0.65 mL/min) with a 0.6 mm diameter needle into CaCl_2_, ZnCl_2_, FeCl_3_, and AlCl_3_ solutions with constant stirring at 25 °C. The salt solutions (170 mM) containing 25% ethanol were placed in a round-bottomed bowl, which allowed for a better distribution of the beads by volume and to avoid beads sticking together in the first seconds of the formation of the gel beads. The distance from the nozzle to the calcium chloride solution was 0.5–1.5 cm. Thus, beads named CaPG, ZnPG, FePG, and AlPG were formed using different cross-linking cations. The pectin gel beads formed were allowed to stand in the respective salt solutions for 24 h and were then washed on the grid three times with 25% aqueous ethanol solution and dried at 25 °C to a constant weight, as described previously [18].

The content of endotoxin was 2.3, 1.5, 2.3, and 2.7 ng/mg in CaPG, ZnPG, FePG, and AlPG, respectively, as measured using the kinetic chromogenic Limulus amoebocyte lysate test (Charles River Endosafe, Inc., Reno, NV, USA). Lipopolysaccharide of *E. coli* 055:B5 (Charles River Endosafe, Inc., Reno, NV, USA) was used for the standard calibration curve.

### 4.2. Characterization of Gel Beads

Images of wet and dried gel beads (*n* = 40) were obtained using an optical microscope (Altami, Russia) equipped with a camera. The projected equivalent diameter of the beads was determined using an image analysis system (ImageJ 1.46r program, National Institutes of Health, Bethesda, MD, USA) with a calibration of 0.024 mm to one pixel.

A compression test of the gel beads was performed using TA-XT Plus Texture Analyzer (Texture Technologies Corp., Stable Micro Systems, Godalming, UK). The wet gel beads were compressed at 25 °C with a 5 mm diameter (P/5) cylinder probe, where the pre- and post-test speed was 5.0 mm/s, and the test speed was 0.5 mm/s for the wet beads and 0.1 mm/s for the dry beads. Gel strength for wet beads was calculated at a distance of 0.5 mm for the dry beads until the destruction of the bead. The detailed procedure was described earlier [19]. The calculations of maximum peaks were performed for ten replicate samples using the Texture Exponent 6.1.4.0 software (Stable Micro Systems, Godalming, UK).

The water content was calculated using the following Equation (1):

(W_W_ − W_D_)/W_W_ × 100%, (1)

where W_W_ and W_D_ represent the weight of the gel beads (*n* = 20–30) before and after drying at 25 °C until constant weight.

### 4.3. Swelling Characterization of Gel Beads

A total of 20 mg of dried gel beads (ca. 30 beads) were incubated in RPMI-1640 medium supplemented with 10% FBS for 1, 6, and 24 h with shaking (100 rpm) in an orbital shaker incubator (Titramax 1000, Heidolph, Germany) at 37 °C. Each RPMI-1640 solution variant was supplemented with 25 mM HEPES to support a constant pH of 7.4. After a predetermined time interval, the projected equivalent diameter of the beads was measured using an optical microscope (Altami, Russia) fitted with a camera and an image analysis system (Altami Studio, Altami, Russia). An image of a linear scale was used for calibration under the same optical conditions. One pixel corresponded to 0.00593 mm. The swelling degree (SD) was calculated using the following Equation (2):

SD% = ((S_1_ − S_0_)/S_0_) × 100, (2)

where S_1_ is the projected equivalent diameter of the bead after a determined incubation time and S_0_ is the initial diameter.

The gel strength of the pectin gel beads was determined after 1, 6, and 24 h of incubation using a compression test as described above.

### 4.4. Protein and LPS Adsorption by Gel Beads

The adsorption of serum proteins was investigated by immersing pectin gel beads in RPMI-1640 medium supplemented with 10% FBS. In all adsorption experiments, dried pectin gel beads were inserted into glass vials and incubated at 37 °C with 100 rpm shaking. Protein solutions without gel beads and the formulations of gel beads free of proteins served as blanks for each experiment. The amount of protein in the sample aliquots was analyzed after pre-defined time intervals of 1, 6, and 24 h of incubation. Upon incubation, samples were centrifuged at 1000× *g* for 20 min at 4 °C (Micro 220 R Hettich Zentrifugen, Tuttlingen, Germany), and the supernatant containing free protein was collected. The free protein content of the supernatants was measured by a Micro BCA Protein Assay Kit (Thermo Scientific™, Waltham, MA, USA). The calibration curve was prepared by measuring varying BSA concentrations in PBS, as the absorbance at 562 nm was measured using a Power wave 200 reader (BioTek Instruments, Santa Clara, CA, USA). Based on this curve, the amount of the adsorbed protein was expressed as μg/mm^2^ and calculated using the following Equation (3):

(C_0_ − C_t_) × V/S, (3)

where C_0_ and C_t_ are the initial concentration of protein and the concentration of protein (mg/L) remaining in solution at different time points after incubation, respectively. V (L) is the total volume of the solution and S (cm^2^) is the total surface area of the pectin gels after removal from the solution.

The adsorption kinetics were estimated as the relative protein concentration [C_t_/C_0_] when the pectin gels were immersed in protein solutions during incubation.

The adsorption of LPS was investigated by immersing pectin gel beads in RPMI-1640 medium supplemented with 10% FBS and 10 μg/mL LPS *E. coli* 055:B5 (Charles River Endosafe, Inc., Reno, NV, USA). In all adsorption experiments, pectin gel beads were inserted into glass vials and incubated at 37 °C with 100 rpm shaking. The amount of free LPS in the sample aliquots was analyzed after pre-defined time intervals of 1, 6, and 24 h of incubation. Upon incubation, samples were centrifuged 1000× *g* for 20 min at 4 °C (Micro 220 R Hettich Zentrifugen, Tuttlingen, Germany), and the supernatant containing free LPS was collected. The concentration of LPS was measured using the kinetic chromogenic Limulus amoebocyte lysate test (Charles River Endosafe, Inc., Reno, NV, USA).

### 4.5. Complement Activation Evaluation

Venous blood was collected from healthy volunteers in vacuum tubes (Improvacuter, Guangzhou Improve Medical Instruments, Guangzhou, China) after obtaining written informed consent. The protocol was approved by the Ethical Committee of the Komi Science Centre of the Russian Academy of Sciences. Complement activation evaluation was performed by measuring the C3a levels using an enzyme-linked immunosorbent assay (ELISA) kit (Human C3a ELISA kit, Hycult Biotech, Uden, The Netherlands) as previously described [18].

### 4.6. Isolation of hPBMCs and Cytokine Production

Venous blood was collected from healthy volunteers in vacuum tubes (Improvacuter, Guangzhou Improve Medical Instruments, Guangzhou, China) after obtaining written informed consent. Isolation of PBMCs from human blood was performed by density gradient centrifugation into Ficoll-1077. Briefly, 300 mL of freshly drawn blood was mixed at a ratio of 1:1 (*v*/*v*) with PBS at room temperature, and 30 mL of the mixture was layered onto 15 mL of Ficoll-1077 in 50 mL tubes. After centrifugation at 500× *g* for 30 min at 20 °C without a break, buffy coats were collected, pooled, resuspended in PBS, and centrifuged at 500× *g* for 20 min at 20 °C without a break. The pellets were resuspended in 60 mL of PBS and again layered onto Ficoll-1077 as described previously. Buffy coats were aspirated, washed with ice-cold PBS, and centrifuged at 500× *g* for 8 min at 4 °C without a break. One half of the harvested PBMCs were cultured overnight in RPMI-1640 supplemented with 1% penicillin/streptomycin and 10 vol% heat-inactivated FBS in 12-well plates in a humidified atmosphere (5 vol% CO_2_, 37 °C).

Isolated hPBMCs (1 × 10^6^/mL) were added to the wells of a 12-well plate and 10 gel beads were added to the cell suspension. LPS was used for stimulation at a final concentration of 2 µg/mL. Cultivation was carried out in 1 mL of RPMI-1640 nutrient medium with 10% heat-inactivated FBS and 1% penicillin/streptomycin under standard conditions (37 °C, 5% CO_2_). Twenty-four hours later, the conditioned medium was removed, aliquoted, and stored at −70 °C. For the cytokine profile, IL-1β, IL-10, and TNF-α were determined using the BD Cytometric bead array (CBA) human soluble protein master Kit.

The human cytokine kit BD Cytometric bead array (CBA) human soluble protein master Kit was used to measure the concentration of interleukin IL-1β, IL-10, and TNF-α in blood human plasma. This array kit provided a mixture of capture beads with distinct fluorescent intensities that were coated with capture antibodies specific for each cytokine. The tests were performed according to the manufacturer’s instructions. About 50 μL of assay beads, 50 μL of detection reagent, and 50 μL of the studied sample or standard were added consecutively to each sample tube and incubated for 3 h at room temperature in the dark. Next, the samples were washed with 1 mL of wash buffer and centrifuged. The supernatant was discarded. The pellet was resuspended in 300 μL of buffer and acquired in the flow cytometer. Samples were measured using a BD FACSCanto II flow cytometer and analyzed with the FCAP Array™ Software (BD Biosciences, Franklin Lakes, NJ, USA). Individual cytokine concentrations were indicated by their fluorescent intensities. Cytokine standards were diluted serially to construct calibration curves to determine the concentration of the analyte.

### 4.7. Measuring the Number of Apoptotic Fibroblasts

A cell culture of human fibroblasts (CellApplications, San Diego, CA, USA, Cat. № 106K-05a) was introduced into the wells of a 12-well plate in the amount of 6–7 × 10^5^/mL, after which 10 gel beads were added to the cell suspension. Cultivation was carried out in 1 mL of Dulbecco’s Modified Eagle Medium (DMEM) with 10% FBS and 1% penicillin/streptomycin under standard conditions (37 °C, 5% CO_2_). A day later, the culture medium was removed, and adherent cells were separated using a tr-EDTA solution, after which the cells were washed three times in 10 mL of PBS and centrifuged for 5 min at 400× *g*.

An FITC Annexin V Apoptosis Detection Kit (BD Pharmingen, San Diego, CA, USA) was used to estimate the number of apoptotic cells. The procedure was carried out according to the manufacturer’s protocol. The cells washed in PBS were resuspended in binding buffer at a concentration of 1 × 10^6^ cells/mL. A total of 100 µL of the solution was transferred to a 5 mL culture tube and 5 µL of FITC Annexin V and 5 µL of propidium iodide were added. The cells were gently vortexed and incubated for 15 min at room temperature in the dark. A total of 400 µL of binding buffer was added to each tube and analyzed by flow cytometry within 1 h.

### 4.8. Statistical Analysis

The results were presented as the arithmetic mean ± standard deviation. One-way ANOVA with Tukey’s honest significance test was applied to determine statistically significant differences in gel characterization, swelling, protein adsorption, and complement activation. ANOVA for repeated measurements was used to determine statistically significant differences in experiments that involved human cell cultures. Values of *p* ≤ 0.05 were considered statistically significant.

## 5. Conclusions

This study revealed the differences in the mechanical properties and swelling behaviors of pectin gel beads cross-linked with Ca^2+^, Zn^2+^, Fe^3+^, and Al^3+^ ions. Trivalent cations (Fe^3+^ and Al^3+^) provided a stronger pectin gel formation than divalent cations (Ca^2+^ and Zn^2+^), probably because of their additional ionic bonding in the egg-box structure. The cations influenced the swelling degree of the dried gel beads in the RPMI-1640 medium in the following order: Zn^2+^ < Ca^2+^ = Fe^3+^ < Al^3+^. The small size and high density of the dry ZnPG beads may explain their lowest swelling, while the greatest swelling of the AlPG beads may be because of the smallest ionic radii of Al^3+^ ions. The type of cross-linking cation was found to influence the in vitro biocompatibility of the pectin gel beads. All the pectin beads adsorbed serum proteins to a low extent and reduced TNF-α and IL-10 production by hPBMCs in response to LPS stimulation. A feature of the biocompatibility of the ZnPG and FePG beads was their serum protein adsorption, which was four times higher than that of the CaPG and AlPG beads. The main finding regarding the ZnPG beads was their extremely high cytotoxicity towards both hPBMCs and fibroblasts, which may limit the use of Zn^2+^-cross-linked pectin gels for engineered cell delivery. The main finding regarding the AlPG beads was their stimulating effect on IL-1β production by hPBMCs, which was consistent with the known use of aluminum salts as immunoadjuvants. For the first time, ionotropic pectin gel was found to increase the number of apoptotic fibroblasts, where the effect of the Al^3+^-cross-linked gel was stronger than that of the Ca^2+^-, Zn^2+^-, and Fe^3+^-cross-linked gels.

Thus, the use of different cross-linking cations changed the properties of the pectin gels, which are important for biocompatibility. Variation in cross-linking cations, besides the variation in the polysaccharide’s structure, is proposed to modify the interaction of pectin-based biomaterials with biological tissues.

## Figures and Tables

**Table 1:** Characterization of gel beads.

Gel Bead	Diameter (mm)	Weight (mg)	S * (mm^2^)	Density (kg/m^3^)	SF **	Gel Strength (N)
Wet CaPG	2.90 ± 0.16 ^ac^	10.70 ± 0.94 ^a^	26.41 ± 2.88 ^a^	857 ± 150 ^a^	0.09 ± 0.05 ^a^	0.28 ± 0.01 ^a^
Wet ZnPG	2.67 ± 0.12 ^b^	10.54 ± 0.92 ^a^	22.47 ± 2.07 ^b^	1067 ± 148 ^b^	0.05 ± 0.02 ^b^	0.48 ± 0.02 ^b^
Wet FePG	2.94 ± 0.22 ^c^	10.11 ± 0.74 ^a^	27.27 ± 4.14 ^a^	785 ± 179 ^ac^	0.09 ± 0.03 ^a^	0.79 ± 0.03 ^c^
Wet AlPG	3.01 ± 0.10 ^c^	10.28 ± 0.93 ^a^	28.51 ± 1.80 ^a^	723 ± 69 ^c^	0.05 ± 0.01 ^b^	0.81 ± 0.07 ^c^
Dried CaPG	1.19 ± 0.10 ^ac^	0.45 ± 0.05 ^a^	4.46 ± 0.77 ^a^	532 ± 128 ^a^	0.09 ± 0.05 ^a^	42.6 ± 2.7 ^a^
Dried ZnPG	0.98 ± 0.04 ^b^	0.49 ± 0.04 ^ab^	3.01 ± 0.26 ^b^	999 ± 127 ^b^	0.06 ± 0.03 ^a^	61.8 ± 2.7 ^b^
Dried FePG	1.15 ± 0.05 ^cd^	0.53 ± 0.03 ^b^	4.13 ± 0.35 ^ac^	674 ± 99 ^c^	0.09 ± 0.04 ^a^	29.2 ± 3.1 ^c^
Dried AlPG	1.10 ± 0.06 ^d^	0.48 ± 0.02 ^ab^	3.82 ± 0.43 ^c^	699 ± 120 ^c^	0.07 ± 0.04 ^a^	27.9 ± 2.8 ^c^

* Surface area; ** sphericity factor. The data are presented as the mean ± standard deviation (SD.) Different letters among means of wet beads indicate significant differences in the same column (*n* = 10, *p* < 0.05). Different letters among means of dried beads indicate significant differences in the same column (*n* = 10, *p* < 0.05).

## Data Availability

The data that support the findings of this study are available from the corresponding author upon reasonable request.

## Supplementary Materials

The following supporting information can be downloaded at: https://www.mdpi.com/article/10.3390/ijms232314789/s1.

## Sample Availability

Samples of the compounds are available from the authors.

## References

[1] Ropartz D., Ralet M.-C., Kontogiorgos V. (2020). Pectin structure. Pectin: Technological and Physiological Properties.

[2] Cao L., Lu W., Mata A., Nishinari K., Fang Y. (2020). Egg-box model-based gelation of alginate and pectin: A review. Carbohydr. Polym..

[3] Lara-Espinoza C., Carvajal-Millán E., Balandran-Quintana R., Lopez-Franco Y., Rascon-Chu A. (2018). Pectin and pectin-based composite materials: Beyond food texture. Molecules.

[4] Minzanova S.T., Mironov V.F., Arkhipova D.M., Khabibullina A.V., Mironova L.G., Zakirova Y.M., Milyukov V.A. (2018). Biological activity and pharmacological application of pectic polysaccharides: A review. Polymers.

[5] Chan S.Y., Choo W.S., Young D.J., Loh X.J. (2017). Pectin as a rheology modifier: Origin, structure, commercial production and rheology. Carbohydr. Polym..

[6] Zhang B., Hu B., Nakauma M., Funami T., Nishinari K., Draget K.I., Phillips G.O., Fang Y. (2018). Modulation of calcium-induced gelation of pectin by oligoguluronate as compared to alginate. Food Res. Int..

[7] Eivazzadeh-Keihan R., Noruzi E.B., Aliabadi H.A.M., Sheikhaleslami S., Akbarzadeh A.R., Hashemi S.M., Gorab M.G., Maleki A., Cohan R.A., Mahdavi M. (2022). Recent advances on biomedical applications of pectin-containing biomaterials. Int. J. Biol. Macromol..

[8] Auriemma G., Cerciello A., Aquino R.P., Del Gaudio P., Fusco B.M., Russo P. (2020). Pectin and Zinc Alginate: The Right Inner/Outer Polymer Combination for Core-Shell Drug Delivery Systems. Pharmaceutics.

[9] Sarioglu E., Kocaaga B.A., Turan D., Batirel S., Guner F.S. (2019). Theophylline-loaded pectin-based hydrogels II. Effect of concentration of initial pectin solution, crosslinker type and cation concentration of external solution on drug release profile. J. Appl. Polym. Sci..

[10] Assifaoui A., Lerbret A., Uyen H.T.D., Neiers F., Chambin O., Loupiac C., Cousin F. (2015). Structural behaviour differences in low methoxy pectin solutions in the presence of divalent cations (Ca^2+^ and Zn^2+^): A process driven by the binding mechanism of the cation with the galacturonate unit. Soft Matter.

[11] Prezotti F.G., Cury B.S.F., Evangelista R.C. (2014). Mucoadhesive beads of gellan gum/pectin intended to controlled delivery of drugs. Carbohydr. Polym..

[12] Günter E.A., Popeyko O.V., Melekhin A.K., Belozerov V.S., Martinson E.A., Litvinets S.G. (2019). Preparation and properties of the pectic gel microparticles based on the Zn^2+^, Fe^3+^ and Al^3+^ cross-linking cations. Int. J. Biol. Macromol..

[13] El-Zahaby S.A., Kassem A.A., El-Kamel A.H. (2014). Formulation and in vitro evaluation of size expanding gastro-retentive systems of levofloxacin hemihydrate. Int. J. Pharm..

[14] Gadalla H.H., El-Gibaly I., Soliman G.M., Mohamed F.A., El-Sayed A.M. (2016). Amidated pectin/sodium carboxymethylcellulose microspheres as a new carrier for colonic drug targeting: Development and optimization by factorial design. Carbohydr. Polym..

[15] Wu X., Sun H., Qin Z., Che P., Yi X., Yu Q., Zhang H., Sun X., Yao F., Li J. (2020). Fully physically crosslinked pectin-based hydrogel with high stretchability and toughness for biomedical application. Int. J. Biol. Macromol..

[16] Giammanco G.E., Sosnofsky C.T., Ostrowski A.D. (2015). Light-responsive iron(III)−polysaccharide coordination hydrogels for controlled delivery. ACS Appl. Mater. Interfaces.

[17] Kulikouskaya V., Kraskouski A., Hileuskaya K., Zhura A., Tratsyak S., Agabekov V. (2019). Fabrication and characterization of pectin-based three-dimensional porous scaffolds suitable for treatment of peritoneal adhesions. J. Biomed. Mater. Res..

[18] Popov S., Paderin N., Khramova D., Kvashninova E., Patova O., Vityazev F. (2022). Swelling, Protein Adsorption, and Biocompatibility In Vitro of Gel Beads Prepared from Pectin of Hogweed *Heracleum sosnówskyi* Manden in Comparison with Gel Beads from Apple Pectin. Int. J. Mol. Sci..

[19] Klopfleisch R., Jung F. (2017). The pathology of the foreign body reaction against biomaterials. J. Biomed. Mater. Res. Part A.

[20] Yang Q., Peng J., Xiao H., Xu X., Qian Z. (2022). Polysaccharide hydrogels: Functionalization, construction and served as scaffold for tissue engineering. Carbohydr. Polym..

[21] Sutar P.B., Mishra R.K., Pal K., Banthia A.K. (2007). Development of pH sensitive polyacrylamide grafted pectin hydrogel for controlled drug delivery system. J. Mater. Sci. Mater. Med..

[22] Abbasi M., Sohail M., Minhas M.U., Khan S., Hussain Z., Mahmood A., Shah S.A., Kousar M. (2019). Novel biodegradable pH-sensitive hydrogels: An efficient controlled release system to manage ulcerative colitis. Int. J. Biol. Macromol..

[23] Bashir S., Hina M., Iqbal J., Rajpar A.H., Mujtaba M.A., Alghamdi N.A., Wageh S., Ramesh K., Ramesh S. (2020). Fundamental concepts of hydrogels: Synthesis, properties, and their applications. Polymers.

[24] Ninan N., Muthiah M., Park I.K., Elain A., Thomas S., Grohens Y. (2013). Pectin/carboxymethyl cellulose/microfibrillated cellulose composite scaffolds for tissue engineering. Carbohydr. Polym..

[25] Kyomugasho C., Gwala S., Christiaens S., Jamsazzadeh Kermani Z., Van Loey A.M., Grauwet T., Hendrickx M.E. (2017). Pectin nanostructure influences pectin-cation interactions and in vitro-bioaccessibility of Ca^2+^, Zn^2+^, Fe^2+^ and Mg^2+^-ions in model systems. Food Hydrocol..

[26] Brash J.L., Horbett T.A., Latour R.A., Tengvall P. (2019). The blood compatibility challenge. Part 2: Protein adsorption phenomena governing blood reactivity. Acta Biomater..

[27] Hedayati M., Neufel M.J., Reynolds M.M., Kipper M.J. (2019). The quest for blood-compatible materials: Recent advances and future technologies. Mater. Sci. Eng. R.

[28] Wang J., Sun H., Li J., Dong D., Zhang Y., Yao F. (2015). Ionic starch-based hydrogels for the prevention of nonspecific proteinadsorption. Carbohydr. Polym..

[29] Isobe T., Kofuji K., Okada K., Fujimori J., Murata M., Shigeyama M., Hanioka N., Murata Y. (2016). Adsorption of histones on natural polysaccharides: The potential as agent for multiple organ failure in sepsis. Int. J. Biol. Macromol..

[30] Gorbet M., Sperling C., Maitz M.F., Siedlecki C.A., Werner C., Sefton M.V. (2019). The blood compatibility challenge. Part 3: Material associated activation of blood cascades and cells. Acta Biomater..

[31] Jin M.-Y., Li M.-Y., Huang R.M., Wu X.-Y., Sun Y.-M., Xu Z.-L. (2021). Structural features and anti-inflammatory properties of pectic polysaccharides: A review. Trends Food Sci. Technol..

[32] O’Hagan D.T., Friedland L.R., Hanon E., Didierlaurent A.M. (2017). Towards an evidence based approach for the development of adjuvanted vaccines. Curr. Opin. Immunol..

[33] Oleszycka E., Moran H.B.T., Tynan G.A., Hearnden C.H., Coutts G., Campbell M., Allan S.M., Scott C.J., Lavelle E.C. (2016). IL-1a and inflammasome-independent IL-1b promote neutrophil infiltration following alum vaccination. FEBS J..

[34] Li H., Nookala S., Re F. (2007). Aluminum hydroxide adjuvants activate caspase-1 and induce IL-1b and IL-18 release. J. Immunol..

[35] Zhang H., Wang P., Yu H., Yu K., Cao Z., Xu F., Yang X., Song M., Li Y. (2018). Aluminum trichloride-induced hippocampal inflammatory lesions are associated with IL-1b-activated IL-1 signaling pathway in developing rats. Chemosphere.

[36] Reinke S., Thakur A., Gartlan C., Bezbradica J.S., Milicic A. (2020). Inflammasome-mediated immunogenicity of clinical and experimental vaccine adjuvants. Vaccines.

