# Supplementary material for: Effect of Cross-Linking Cations on In Vitro Biocompatibility of Apple Pectin Gel Beads
Source: Int J Mol Sci. 2022 Nov 26;23(23):14789. doi: 10.3390/ijms232314789 (PMC9741146; doi:10.3390/ijms232314789)

Supplementary materials

Figure S1. Results of cytometric live (green) /dead (red) test in hPBMCs incubated with pectin gel beads.

(A) Control

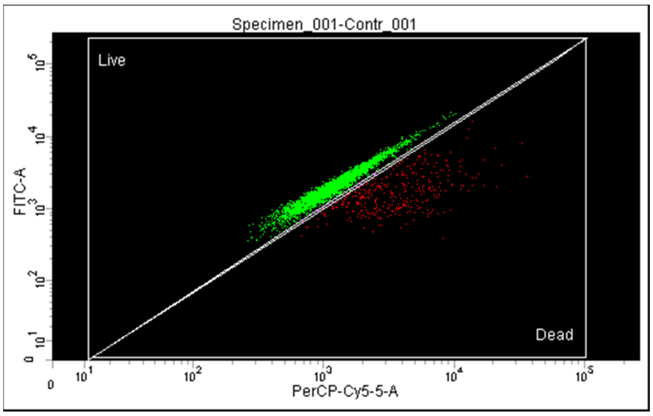

(B) CaPG beads

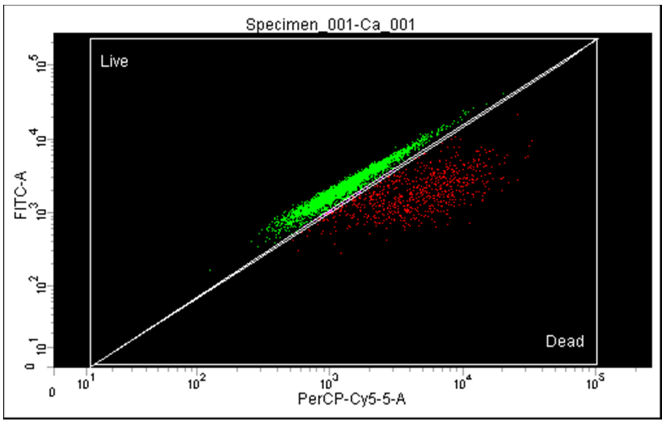

(C) ZnPG beads

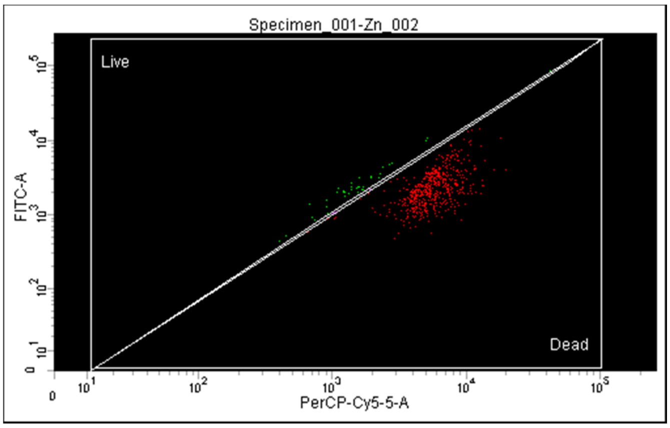

(D) FePG beads

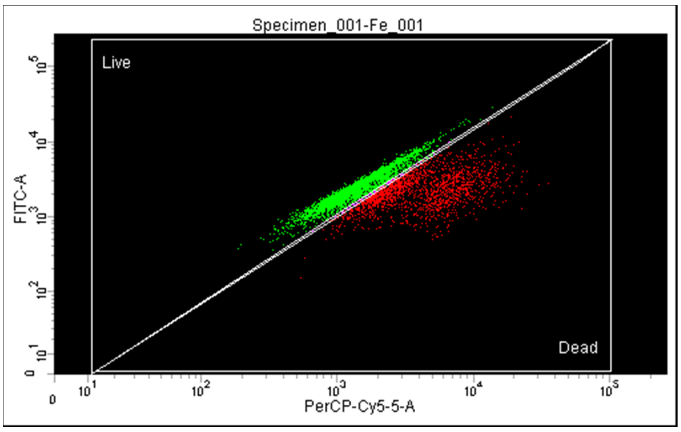

(E) AlPG beads

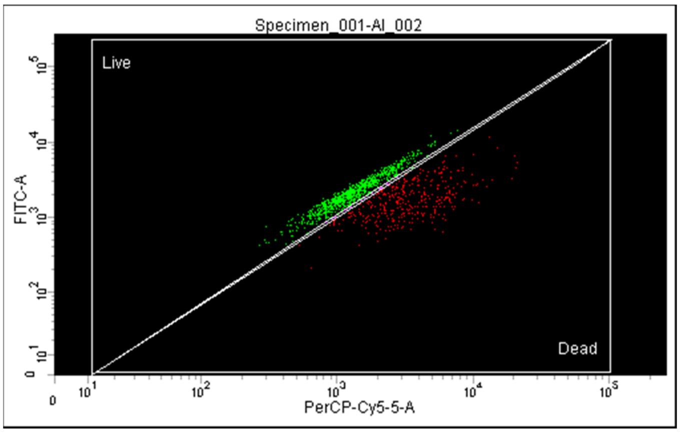

Figure S2. Monolayer of human fibroblasts incubated with pectin gel beads.

(A) Control

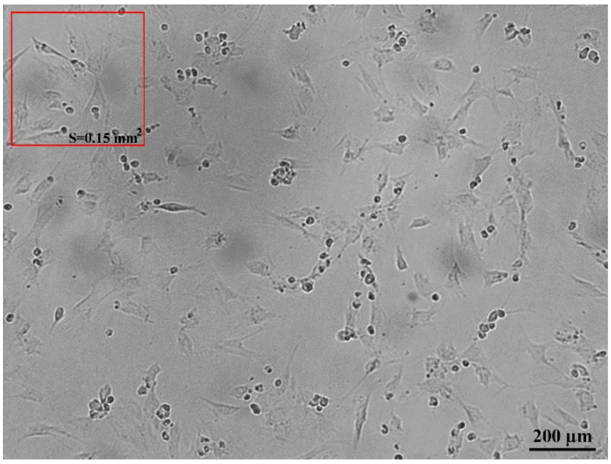

(B) CaPG beads

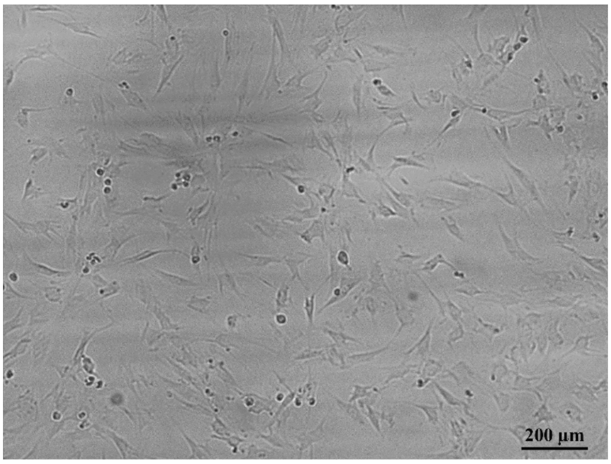

(C) ZnPG beads

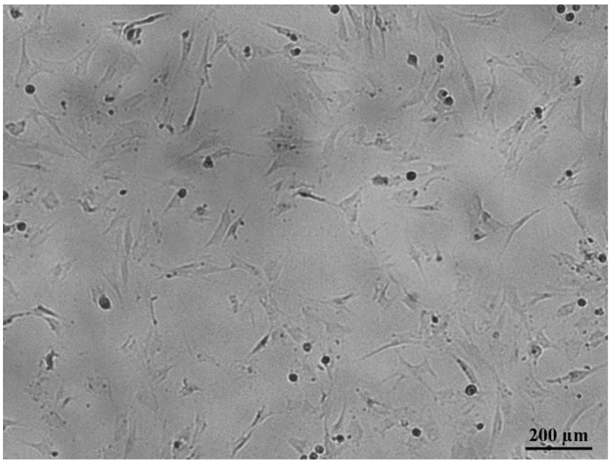

(D) FePG beads

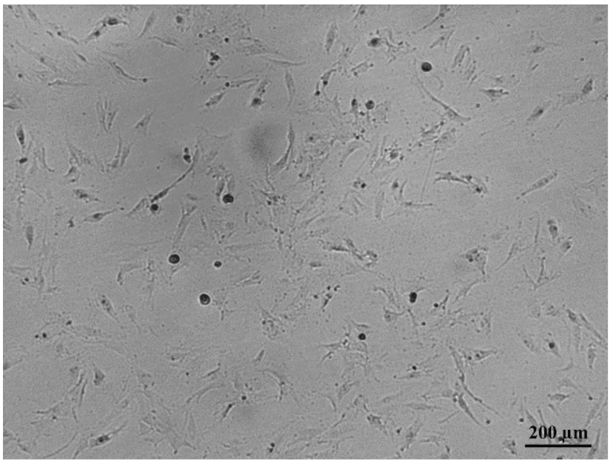

(E) AlPG beads

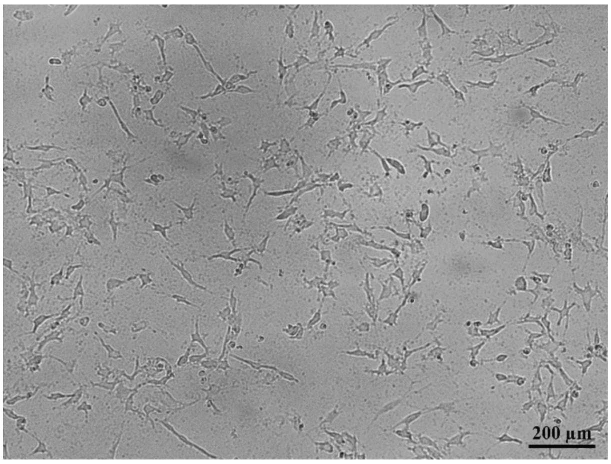

Supplement: Supplementary file 1 [file ijms-23-14789-s001.zip › ijms-2031916-supplementary.pdf]
